# The mammalian gene function resource: the international knockout mouse consortium

**DOI:** 10.1007/s00335-012-9422-2

**Published:** 2012-09-12

**Authors:** Allan Bradley, Konstantinos Anastassiadis, Abdelkader Ayadi, James F. Battey, Cindy Bell, Marie-Christine Birling, Joanna Bottomley, Steve D. Brown, Antje Bürger, Carol J. Bult, Wendy Bushell, Francis S. Collins, Christian Desaintes, Brendan Doe, Aris Economides, Janan T. Eppig, Richard H. Finnell, Colin Fletcher, Martin Fray, David Frendewey, Roland H. Friedel, Frank G. Grosveld, Jens Hansen, Yann Hérault, Geoffrey Hicks, Andreas Hörlein, Richard Houghton, Martin Hrabé de Angelis, Danny Huylebroeck, Vivek Iyer, Pieter J. de Jong, James A. Kadin, Cornelia Kaloff, Karen Kennedy, Manousos Koutsourakis, K. C. Kent Lloyd, Susan Marschall, Jeremy Mason, Colin McKerlie, Michael P. McLeod, Harald von Melchner, Mark Moore, Alejandro O. Mujica, Andras Nagy, Mikhail Nefedov, Lauryl M. Nutter, Guillaume Pavlovic, Jane L. Peterson, Jonathan Pollock, Ramiro Ramirez-Solis, Derrick E. Rancourt, Marcello Raspa, Jacques E. Remacle, Martin Ringwald, Barry Rosen, Nadia Rosenthal, Janet Rossant, Patricia Ruiz Noppinger, Ed Ryder, Joel Zupicich Schick, Frank Schnütgen, Paul Schofield, Claudia Seisenberger, Mohammed Selloum, Elizabeth M. Simpson, William C. Skarnes, Damian Smedley, William L. Stanford, A. Francis Stewart, Kevin Stone, Kate Swan, Hamsa Tadepally, Lydia Teboul, Glauco P. Tocchini-Valentini, David Valenzuela, Anthony P. West, Ken-ichi Yamamura, Yuko Yoshinaga, Wolfgang Wurst

**Affiliations:** 1 The Wellcome Trust Sanger Institute, Wellcome Trust Genome Campus, Hinxton, Cambridge, CB10 1HH UK; 2 Biotechnology Center (BIOTEC) of the Technische Universität Dresden, 01307 Dresden, Germany; 3 Institut Clinique de la Souris and Institut de Génétique et de Biologie Moléculaire et Cellulaire, 67404 Illkirch Cedex, France; 4 National Institute on Deafness and Other Communication Disorders (NIH), Bethesda, MD 20892 USA; 5 Genome Canada, Ottawa, ON K2P 1P1 Canada; 6 Mammalian Genetics Unit, MRC Harwell, Harwell Science and Innovation Campus, Oxfordshire, OX11 0RD UK; 7 Institute of Developmental Genetics, Helmholtz Zentrum München, Technische Universität München, 85764 Neuherberg, Germany; 8 The Jackson Laboratory, Bar Harbor, ME 04609 USA; 9 National Institutes of Health, Bethesda, MD 20892 USA; 10 Infectious Diseases and Public Health, European Commission, DG Research & Innovation, 1049 Brussels, Belgium; 11 Istituto di Biologia Cellulare, Consiglio Nazionale delle Ricerche (CNR), Monterotondo-Scalo, 00015 Rome, Italy; 12 Velocigene Division, Regeneron Pharmaceuticals Inc., Tarrytown, NY 10591 USA; 13 The Texas A&M Institute for Genomic Medicine, College Station, TX 77843-4485 USA; 14 University of Texas at Austin, Austin, TX 78712 USA; 15 National Institutes of Health, Bethesda, MD 20205 USA; 16 Department of Cell Biology, Center of Biomedical Genetics, Erasmus University Medical Center, 3015 GE Rotterdam, The Netherlands; 17 Manitoba Institute of Cell Biology, University of Manitoba, Winnipeg, MB R3E OV9 Canada; 18 Institute of Experimental Genetics, Helmholtz Zentrum München, 85764 Neuherberg, Germany; 19 Department of Development and Regeneration, Faculty of Medicine, University of Leuven (KU Leuven), 3000 Leuven, Belgium; 20 Children’s Hospital Oakland Research Institute (CHORI), Oakland, CA 94609 USA; 21 Mouse Biology Program, School of Veterinary Medicine, University of California, Davis, CA 95616 USA; 22 Research Institute, The Hospital for Sick Children, SickKids Foundation, Toronto, ON M5G2L3 Canada; 23 Department of Molecular Haematology, University of Frankfurt Medical School, 60590 Frankfurt am Main, Germany; 25 Samuel Lunenfeld Research Institute, Mount Sinai Hospital, Joseph and Wolf Lebovic Health Complex, Toronto, ON M5G 1X5 Canada; 26 Division of Basic Neuroscience and Research, National Institute of Drug Abuse (NIDA), Bethesda, MD 20892-0001 USA; 27 Department of Biochemistry and Molecular Biology, University of Calgary, Calgary, AB T2N 1N4 Canada; 28 European Molecular Biology Laboratory (EMBL), Monterotondo, 00015 Rome, Italy; 29 Centre for Cardiovascular Research, Department of Vertebrate Genomics, Charité, 10115 Berlin, Germany; 30 Department of Physiology, Development and Neuroscience, University of Cambridge, Cambridge, CB2 3EG UK; 31 Department of Medical Genetics, Centre for Molecular Medicine and Therapeutics at the Child & Family Research Institute, University of British Columbia, Vancouver, BC V5Z 4H4 Canada; 32 European Bioinformatics Institute (EBI), Hinxton, Cambridge, CB10 1ST UK; 33 The Ottawa Hospital Research Institute, Ottawa, ON K1N 6N5 Canada; 34 Division of Developmental Genetics, Center for Animal Resources and Development, Institute of Resource Development and Analysis, Kumamoto University, Kumamoto, 860-0811 Japan; 35 Icahn Medical Institute, The Mount Sinai Hospital, New York, NY 10029 USA; 36 Max-Planck-Institute of Psychiatry, 80804 Munich, Germany; 37 Deutsches Zentrum fuer Neurodegenerative Erkrankungen e.V. (DZNE) Site Munich, 80336 Munich, Germany

## Abstract

In 2007, the International Knockout Mouse Consortium (IKMC) made the ambitious promise to generate mutations in virtually every protein-coding gene of the mouse genome in a concerted worldwide action. Now, 5 years later, the IKMC members have developed high-throughput gene trapping and, in particular, gene-targeting pipelines and generated more than 17,400 mutant murine embryonic stem (ES) cell clones and more than 1,700 mutant mouse strains, most of them conditional. A common IKMC web portal (www.knockoutmouse.org) has been established, allowing easy access to this unparalleled biological resource. The IKMC materials considerably enhance functional gene annotation of the mammalian genome and will have a major impact on future biomedical research.

## Introduction

Annotation of the human and mouse genomes has identified more than 20,000 protein-coding genes and more than 3,000 noncoding RNA genes. Together, these genes orchestrate the development and function of the organism from fertilization through embryogenesis to adult life. Despite the dramatic increase in knowledge of variation in human genomes of healthy and diseased individuals, the normal functions of common forms of most genes are still unknown and consequently the disease significance of rare variants remains obscure as well.

To determine gene function, mutation of those genes is required in model organisms. The mouse has long been regarded as ideal for this purpose. Conservation of most aspects of mammalian development, anatomy, metabolism, and physiology between humans and mice is underscored by strong one-to-one orthologous relationships between genes of the two species. Conservation of gene function is strongly supported by similar phenotypic consequences of complete or partial loss-of-function mutations in orthologous genes in both species and by functional replaceability of mouse genes by their human counterparts (Wallace et al. 2007).

To provide a platform for addressing vertebrate gene function on a large scale, the research community came together to establish a genome-wide genetic resource of mouse mutants (Austin et al. 2004; Auwerx et al. 2004). The consensus was that the future currency of this biological resource should be based on ES cells, which can be readily transferred between laboratories and across international boundaries. It was also felt that the most desirable alleles would be those generated by gene targeting. Bespoke designs for each gene would accommodate each gene’s unique structural attributes and take account of adjacent genomic features. Uncertainty in the scalability of gene-targeting technology coupled with the availability of several gene-trap libraries and the speed with which additional mutant alleles could be generated by gene-trapping methods resulted in agreement that the resource should be generated initially by using both gene-targeting and gene-trapping technologies.

Thus, the vision emerged of a core public archive of ES cell clones on a single uniform genetic background, each clone carrying an engineered mutation in a different gene. To extract biological insights from this resource, individual ES cell clones would be converted into mice by individual investigators and organized programs.

To deliver the ES cell resource toward this vision of functional annotation, four international programs in Europe and North America were established with the goal of achieving saturation mutagenesis of the mouse genome: EUCOMM, KOMP, NorCOMM, and TIGM (see Table 3). These programs were the founding members of the International Knockout Mouse Consortium (IKMC), fostering groups to work together in a highly coordinated, standardized manner, to share technologies, to maximize output, and to largely avoid duplication of effort (Collins et al. 2007). The IKMC consortium has generated mainly conditional but also constitutive mutations, with the former class of mutations facilitating tissue-specific assessment of gene function at desired time points, especially in situations where an essential requirement of a gene product in one context can exclude analysis in another.

## IKMC technology

The IKMC mutant ES cell resources have been developed for the most part in a C57BL/6N genetic background using cell lines that have achieved clonal germline transmission rates of up to 80 % (Pettitt et al. 2009). Mutations were generated initially by using both gene-trapping and gene-targeting technologies. However, the greater utility and desirability of targeted alleles, designed and generated with nucleotide precision, led to phasing out of gene trapping as the efficiency of the high-throughput gene-targeting pipelines became established. Progressive improvements in mouse genome annotation, computational targeting vector design, and 96-well recombineering protocols as well as high efficiencies of gene targeting have facilitated the rapid construction of targeted ES cell clones at unprecedented rates (Skarnes et al. 2011).

**Fig. 1:**
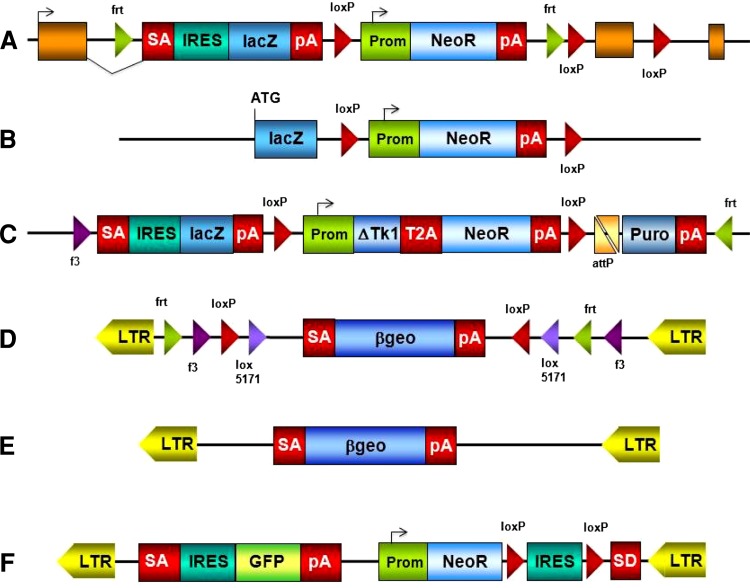
Vectors used by the IKMC: targeting vectors: **a** EUCOMM/KOMP-CSD knockout-first allele; **b** KOMP-Regeneron null allele generating large deletions; **c** NorCOMM promoter-driven targeting vector. Most commonly used trapping vectors: **d** conditional EUCOMM vector rsFlipROSAβgeo*; **e** TIGM vector VICTR76; **f** NorCOMM vector UPA

The alleles generated by IKMC members are *lacZ* tagged and are either null/conditional or null/deletion alleles (Fig. 1). The largest category of targeted clones in the resource contains an allele design known as “knockout-first” from which conditional alleles can be established following exposure to a site-specific recombinase. A conditional allele is created by the deletion of a critical exon which is flanked by loxP sites. Critical exons are those that (1) when deleted, shift the reading frame, (2) are common to all known isoforms, and (3) are contained in the first 50 % of the coding region. Conditional alleles are also amenable to further modification by recombinase-mediated cassette exchange (RMCE), which can be used to insert other coding sequences into these alleles (Osterwalder et al. 2010; Schnütgen et al. 2011). The other major class of mutations in the resource comprises *lacZ*-tagged nulls, constructed as large deletions that are not amenable to further modification (Valenzuela et al. 2003).

## IKMC ES cell and mouse resources

Currently, the IKMC ES cell resource contains targeted and trapped alleles for 17,473 unique protein-coding genes. The targeted ES cell resource contains mutations in 13,840 unique genes of which 10,100 are null/conditional. The ES cell gene-trap resource contains mutations in 11,539 unique genes of which 4,414 are conditional (Table 1, www.knockoutmouse.org). Due to the random nature of gene trapping, there is some overlap and redundancy between the resources. In general, the IKMC has generated a median of four independent clones per gene. This redundancy not only helps to assure germline transmission but also provides significant allelic diversity (e.g., conditional or nonconditional alleles, different insertion sites, and vector designs). The use of 129 Sv ES cells early in the project for the gene-trap resource also provides the option of studying mutations in the same gene in two different genetic backgrounds for 4,600 genes in the resource.

**Table 1:** IKMC progress (as of February 2012)

Total genes	KOMP		EUCOMM	NorCOMM	TIGM
	CSD	Regeneron			
Targeting vectors	6,577	5,905	8,379	916	–
Targeted ES cells	5,244	4,196	6,887	609	–
Trapped ES cells			4,414	3,595	9,390
Mutant mice	409	359	647	42	252

The process of converting IKMC ES cell resources into mice was initiated alongside the gene-targeting and -trapping pipelines as a systematic quality control measure and also for phenotyping purposes. So far, 1,709 mutant mouse lines have been established by all the IKMC members (Table 1). Generation of mice at scale in addition has taken place in organized efforts in a few centers, coupled with systematic phenotyping programs such as the EC-supported EUMODIC Project and the Wellcome Trust Sanger Institute’s Mouse Genetics Programme (MGP) (Table 3). As of July 2012, from both projects’ databases, i.e., www.europhenome.org and www.sanger.ac.uk/mouseportal, already 919 targeted lines can be identified that have been phenotypically examined. Of these, 652 have an annotated phenotype.

## IKMC repositories and distribution

Repositories have an essential function to secure and maintain the investment in the resource for future generations of scientists. Repositories have the obligation to ensure and preserve the quality of the resource and to act as honest brokers with efficient, unrestricted, and unencumbered distribution to third-party users.

The distribution of IKMC vectors and ES cells takes place through several repositories in Europe, the US, and Canada (EuMMCR, KOMP, TIGM, and CMMR, Tables 2, 3). The repositories conduct stringent quality control on the vectors and ES cell clones prior to distribution to ensure the integrity of each allele. They currently distribute nonoverlapping sectors of the IKMC resource to a global user group. Altogether, these centers have already distributed 1,029 targeting vectors and 4,126 mutant ES cell alleles.

**Table 2:** IKMC reagents distributed (as of February 2012)

Type of material	KOMP	EuMMCR/EMMA	CMMR	TIGM
Targeting vectors	677	346	6	–
Mutant ES cell clones (alleles)	3,798 (1,345)	5,041 (1,867)	520 (311)	603 (603)
Mutant mice (alleles)	430 (310)	600 (530)	19 (15)	252 (252)

**Table 3:** Relevant IKMC web sites

Function	Acronym	Full name	Web address
PORTAL	IKMC	International Knockout Mouse Consortium	www.knockoutmouse.org
PRODUCTION PIPELINE	EUCOMM	European Conditional Mouse Mutagenesis Program	www.knockoutmouse.org/about/eucomm
	EUCOMMTOOLS	EUCOMM: Tools for Functional Annotation of the Mouse Genome	www.knockoutmouse.org/about/eucommtools
	KOMP	Knockout Mouse Project	www.knockoutmouse.org/about/komp
	NorCOMM	North American Conditional Mouse Mutagenesis Project	www.norcomm.org
	Sanger	Sanger Institute Mouse Genetics Program (MGP)	www.sanger.ac.uk/mouseportal/
	TIGM	Texas A&M Institute for Genomic Medicine	www.tigm.org
GENETIC TOOL BOX	Create	Coordination of Cre resources	www.creline.org
	MGI Cre	Recombinase (Cre) Portal	www.creportal.org
	Jax Cre	Jax Cre repository	www.cre.jax.org
	GENSAT Cre	GENSAT Cre mice	www.gensat.org/cre.jsp
	IMSR Cre	IMSR Cre archive	www.findmice.org/fetch?page=imsrSummary&query=cre+recombinase
	Cre-X-Mice	Cre-X-Mice	http://nagy.mshri.on.ca/cre_new/index.php
	ICS-CreZoo	ICS CreER^T2^ Zoo	www.ics-mci.fr/crezoo.html
	CanEuCre	Brain specific Cre mice	www.caneucre.org
	TgDb	Transgenic Mice Database (TgDb)	www.bioit.fleming.gr/tgdb/
DISSEMINATION	EuMMCR	European Mouse Mutant Cell Repository	www.eummcr.org
	KOMP	KOMP Repository	www.komp.org
	CMMR	Canadian Mouse Mutant Repository	www.cmmr.ca
	TIGM	Texas A&M Institute for Genomic Medicine	www.tigm.org
	JAX	Jackson Laboratory Mice and Services	www.jaxmice.jax.org
	EMMA	European Mouse Mutant Archive	www.emmanet.org
	MMRRC	Mutant Mouse Regional Resource Center	www.mmrrc.org
PHENOTYPING	Europhenome	Mouse Phenotyping Resource	www.europhenome.org
	EuMODIC	European Mouse Disease Clinic	www.eumodic.org
	TCP	Toronto Centre for Phenogenomics	www.phenogenomics.ca
	KOMP	KOMP Phenotyping Pilot	http://kompphenotype.org
	IMPC	International Mouse Phenotyping Consortium	http://mousephenotype.org

The IKMC mice are distributed through EMMA, MMRRC/KOMP, TIGM, and CMMR, the European, US, and Canadian mouse repositories, respectively (Tables 2, 3). To date, of the 1,709 mutated mouse lines, 1,107 (65 %) have been distributed outside of the center in which they were initially produced (Table 2). The dominant impact of IKMC resources on the activity of these repositories is already apparent, e.g., more than 50 % of mice distributed by EMMA were derived from IKMC resources. IKMC mice are also being generated and analyzed in distributed activities by hundreds of individual specialist laboratories worldwide, which to date have cumulatively received 4,126 mutant ES cell alleles from the repositories.

In summary, to date already 6,262 mutant alleles have been distributed worldwide as vectors, ES cells, or mice (about 35 % of IKMC alleles available), and the international requests are still increasing.

## IKMC web portal

The integrated, public IKMC web portal (http://www.knockoutmouse.org) summarizes the IKMC progress and enables researchers to obtain IKMC genetic resources from designated repositories. It links to IKMC members’ web pages and to related genetic resources such as those available from the International Gene Trap Consortium (IGTC) and the International Mouse Strain Resource (IMSR) (Table 3) (Ringwald et al. 2011). The IKMC alleles are displayed on the Ensembl and UCSC genome browsers with standardized allele identification registered in Mouse Genome Informatics (MGI, www.informatics.jax.org) database.

The IKMC web portal supports use of the genetic resources through provision of detailed molecular structures of mutant alleles. Researchers may nominate genes of interest for prioritization if targeted mutations are not yet available, although the current coverage of 87 % of the protein-coding genes provides a high chance that a useful allele has already been generated.

## IKMC and beyond

The IKMC ES cell resource was envisaged as an essential stepping stone required for the efficient generation of mouse mutants at scale. This resource is nearing completion and is already being accessed by novel large-scale programs to generate mice for phenotypic analysis. An organized effort, the International Mouse Phenotyping Consortium (IMPC), has just been launched (Table 3). Many centers worldwide will contribute to this effort, ultimately generating and analyzing more than 15,000 mouse mutants based on the IKMC resource over the next decade, establishing what will constitute a functional Encyclopedia of the Mammalian Genome.

The IKMC resource, although very comprehensive, still remains incomplete. Ongoing efforts, performed mainly by the EUCOMMTools project (Table 3), will improve genome coverage and the quality of the mutant alleles in the resource over the next 3 years. This will include replacing gene-trap alleles with targeted ones and generating conditional alleles when only a null allele is available. The resource will also continue to expand to cover other classes of genes such as noncoding RNAs.

The IKMC resource includes both vectors and ES cells that can be further modified by RMCE to insert different coding sequences into any conditional mutant IKMC allele such as reporter genes, recombinases, and human orthologs or, e.g., an allele’s missense mutations. In an era in which an extraordinary amount of human variation sequence is becoming available, functional assessment of variants will require analysis under physiological levels of expression and regulation, most reliably achieved by insertion into the mouse orthologous locus.

The examination of a null allele is the requisite first step in order to establish the ground state (null phenotype) of any gene function in the genome. Subsequently, it is desirable to conduct more focused analysis by controlling the generation of the mutation temporally and spatially. The conditional sector of the resource is designed for use with the recombinase Cre, for which a number of transgenic mouse lines are available. The number and characterization level of these lines will increase with various initiatives currently underway [like EUCOMMTools, CanEuCre, and the NorCOMM successor project NorCOMM2LS (Table 3)], and will eventually cover the majority of embryonic and adult tissues and cell types.

Phenotypic analysis of mutant mouse lines generated from IKMC resources enables a better understanding of complex biology such as whole organism physiology, behavior, and adult tissue integrity. In addition, biological insights can also be gained from analysis of cellular phenotypes in culture. IKMC resources facilitate the generation of homozygous mutant ES cells by retargeting the second allele in vitro.

Genetic manipulation of mouse ES cells has reached such a degree of efficiency and sophistication that ES cells can be generated with virtually any genetic change. The success of these technologies provides the groundwork for extension to other mammalian species such as rat, and to human ES and induced pluripotent stem (iPS) cells.
